# Diets, Foods and Food Components’ Effect on Dyslipidemia

**DOI:** 10.3390/nu13030741

**Published:** 2021-02-26

**Authors:** Federica Fogacci, Claudio Borghi, Arrigo F. G. Cicero

**Affiliations:** 1 Medical and Surgical Science Department, University of Bologna, 40138 Bologna, Italy; claudio.borghi@unibo.it (C.B.); arrigo.cicero@unibo.it (A.F.G.C.); 2 IRCCS Azienda Ospedaliero, Universitaria di Bologna, 40138 Bologna, Italy; 3 Italian Nutraceutical Society (SINut), 40138 Bologna, Italy

Hypercholesterolemia is a well-known independent risk factor for cardiovascular disease and a recognized target of pharmacological therapeutic agents in both primary and secondary prevention [1]. There is increasing interest in the use of natural lipid-lowering compounds that may delay or circumvent drug therapy. These compounds could be included as part of a diet pattern, as single foods or as food components transformed into dietary supplements [2].

To date, there is a strong evidence showing that dietary factors are able to influence atherogenesis. In particular, the Mediterranean diet is particularly rich in active vegetable compounds, contributing to its positive effect on human health [3]. However, other active compounds could be isolated and concentrated from non-nutrient vegetable sources, such as medicinal plants [4]. Finally, natural dietary and non-dietary lipid-lowering compound could have pleiotropic effect on lipids and on other cardiovascular risk factors, for instance increasing the cholesterol resistance to oxidative stress, reducing microinflammation, improving the endothelial health, etc. [5].

Based on this background, the volume entitled “*Diets, Foods and Food Components’ Effect on Dyslipidemia*” samples the contributions of a number of recognized experts in the field.

An interesting review summarizes the number of natural compounds reducing the plasma level of Proprotein convertase subtilisin/kexin type 9 (PCSK9), a key enzyme in the metabolism of LDL-cholesterol receptors on the liver cell surface [6]. 

Trautwein and McKay reviewed the effect of plant-based diet on dyslipidemia and cardiovascular risk [7]. Carresi et al. considered the potential effect of bergamot polyphenolic fraction on the prevention of metabolic syndrome [8] and Santos et al. critically appraised the efficacy of plant-derived omega-3 polyunsaturated fatty acids from foodstuffs and supplements upon lipid profile and several cardiometabolic markers [9].

A range of preclinical evidence highlights the antiobesity effect of *Chrysanthemum morifolium* [10], the antioxidant effect of red propolis [11], the cholesterol-lowering and hepatoprotective effects of black raspberry [12], the effect of blackcurrant in preventing dyslipidemia and hepatic steatosis [13], the antioxidant activity of polyphenon-60 from green tea in chitosan microspheres [14], and the combined effect of omega-3 fatty acids and glibenclamide on abnormal lipid profile, increased blood glucose and impaired liver and kidney functions [15].

New epidemiological evidence supports a link between different dietary patterns [16], charbohydrate intake [17], fructose intake [18], vitamin B12 serum levels [19] and lipid profile and other markers of cardiovascular disease risk. Findings from the randomized clinical trials included in the Special Issue support the use of new nutraceutical formulations with lipid-lowering effects. The BELT (Beta-glucan Effects on Lipid profile, glycemia and inTestinal health) study evaluated the medium-term effects of dietary supplementation with oat fibers in a sample of healthy subjects [20], and the NATCOL (NutrAceuTical COmbination on Low-density lipoprotein cholesterol) study tested the effect of dietary supplementation with Levelip Duo^®^ on low-density lipoprotein cholesterol concentrations and lipid profile in subjects with suboptimal blood cholesterol levels [21]. Finally, a systematic review and meta-analysis of randomized controlled clinical studies included in the Special Issue supports the use of Armolipid Plus^®^ in clinical practice [22].

In this context, the volume is a source of new knowledge on the effect of diets, foods and food components on dyslipidemia.
